# Efficacy of stem cells therapy for Crohn’s fistula: a meta-analysis and systematic review

**DOI:** 10.1186/s13287-020-02095-7

**Published:** 2021-01-07

**Authors:** Yantian Cao, Qi Su, Bangjie Zhang, Fangfang Shen, Shaoshan Li

**Affiliations:** 1 Department of Gastroenterology, The Third Affiliated Hospital of Xinxiang Medical University, Hua Lan Avenue, Xinxiang, 453003 Henan Province China; 2 Department of General surgery, The Third Affiliated Hospital of Xinxiang Medical University, Hua Lan Avenue, Xinxiang, 453003 Henan Province China; 3 The Key Laboratory for Tumor Translational Medicine, The Third Affiliated Hospital of Xinxiang Medical University, Hua Lan Avenue, Xinxiang, 453003 Henan Province China

**Keywords:** Stem cells, Crohn’s fistula, Healing rate, Treatment-related adverse events

## Abstract

**Background:**

Fistulas have puzzled us all the time and stem cell therapy for it is still in its infancy. We conducted a meta-analysis and systematic review to evaluate the efficacy of stem cells and its potential mechanisms in the management of Crohn’s fistula.

**Methods:**

Electronic databases were searched comprehensively for studies reporting the efficacy and safety of stem cells in patients with any form of Crohn’s fistula. A random-effects model was used, and all outcomes were calculated by SPSS 24.0.

**Results:**

Twenty-nine articles with 1252 patients were included. It showed that stem cell group had a higher rate of fistula healing compared to placebo group in patients of Crohn’s fistula (61.75% vs 40.46%, OR 2.21, 95% CI 1.19 to 4.11, *P < 0*.*05*). 3 × 10^7^ cells/mL stem cell (SC) group had an advantage in fistula healing rate with 71.0% compared to other doses group of stem cells (RR 1.3, 95% CI 0.76 to 2.22). And the healing rates of patients with perianal and transsphincteric fistulas (77.95%, 76.41%) were higher than those with rectovaginal fistulas. It was an amazing phenomenon that CDAI and PDAI scores occurred an obviously transient rise with the use of stem cells after 1 month (both of *P < 0*.*05*), while they returned to the baseline level by giving stem cells 3 months later. Furthermore, the incidence rate of treatment-related adverse events in the stem cell group was significantly lower than in the placebo group (RR 0.58, 95% CI 0.30 to 1.14).

**Conclusions:**

Our study has highlighted that stem cells was a promising method in the treatment of Crohn’s fistula based on its higher efficacy and lower incidence of adverse events, especially ADSCs and Cx601. While it also needs more clinical and pre-clinical studies to strengthen evidences in the future.

## Introduction

Crohn’s disease (CD) is a chronic, idiopathic inflammation of the whole gastrointestinal tract [1–3]. One of its common and baffling complications is fistula, an abnormal, tunnel-like connection between bowel and nearby epithelial surfaces [4, 5]. And its incidence rate is up to 17 to 50% among CD patients in worldwide [6], in which, 18 to 50% of patients with Crohn’s fistula require surgical removal treatment according to population-based studies [7, 8]. And it is also characterized by facing a notoriously difficult surgical challenge of surgeons’ skills due to its high recurrence [9]. Moreover, since patients with Crohn’s fistula have suffered persistent fecal or urinary seepage, pain, and infection, Crohn’s fistula brought people severe and diverse somatic and social issues, such as sleep disturbance, sexual dysfunction, and bad personal hygiene which reduced the quality of life ultimately [10–12]. Previous studies have shown that impaired local immune system, mucosal and transmural inflammation, luminal bacteria disorder, and hereditary susceptibility might play important roles [13–17]. Yet the specific mechanism is uncertain.

According to the high incidence, mortality, and disability rate, a large amount of researches about treatment of Crohn’s fistula are on the way. The current conventional clinical managements include medications, endoscopic therapy, and surgery. Medications mainly include conventional anti-inflammatory agents such as 5-aminosalicylic acids (5-ASA) and systemic/local corticosteroids, as well as immunomodulators (azathioprine, 6-mercaptopurine (6-MP), cyclosporine, methotrexate, and anti-tumor necrosis factors (TNFs)) [18, 19]. Unfortunately, they have low rates of fistula closure like antibiotics of 21–48%, thiopurines of 20–40%, and infliximab of 23% [11] and have a recurrence rate of about 40.9% [20]. What is more, taking these drugs could lead to various adverse effects companied by lower compliance of patients [21–23]. With the development of diversities of endoscopy, they aimed at finding lesions and carrying drugs to the target in the patients of Crohn’s fistula [24–27]. Its functions are limited. By comparison, surgical removal or drainage of fistulas is the mainstay option to eliminate them at present [28, 29]. But its incidence of postoperative complications was up to 50%, supported by Patil and Cross [30]. And a multicenter trial reported that improvement of inflammatory bowel disease questionnaire (IBDQ) and the MOS item short from health survey score (SF-36) had no difference in the laparoscopic ileocecal resection group of fistula and in the infliximab group (178.10 vs 172.00, 112.10 vs 106.50, *P > 0*.*05*, [31]). There was no doubt that combination of medical and surgical treatment had been superior to single medicine treatment in fistula closure (53% vs 43%, *P < 0*.*05*) [16].

Stem cell therapy (SCT) has emerged as a novel significant approach to improve the clinical remission and response in a number of inflammatory diseases and tissue regeneration due to its properties of immunoregulatory and multiple differentiation potential by releasing various mediators, including immunosuppressive molecules, growth factors, exosomes, chemokines, complement components, and multiple metabolites [32–34]. As the American Society of Colon and Rectal Surgeons said, the goals of Crohn’s fistula management are alleviation of symptoms by eradication of the fistula, prevention of recurrence and preservation of sphincter integrity and continence [35]. Subsequently, SCT is envisaged as an effective alternative to patients of Crohn’s fistula. Some animal studies had already demonstrated that bone marrow-derived stem cells (BMSCs) were able to repair injured intestinal mucosa through downregulating the immune function of T lymphocytes [36]. Meanwhile, García-Olmo et al. conducted a phase I clinical trial in 2005, showing that the fistula healing rate (HR) reached to 60% with the use of adipose-derived mesenchymal stem cells (ADSCs) [37]. Since then, the application of ADSCs in Crohn’s fistula has been explored increasingly due to its advantages of easy acquisition, low cost-saving of adipose tissue, and minimal invasion [32, 38]. Our previous review also reported that approximately 62.52% of patients with Crohn’s fistula achieved complete clinical remission by using stem cells [39]. Until 2016, the study of Panés et al. (a randomized, double-blind, parallel-group, placebo-controlled trial) demonstrated that local allogeneic darvadstrocel (Cx601, Alofisel, Takeda) administration could be an effective and safe treatment for complex perianal fistulas in patients with Crohn’s disease. Expanded allogeneic adipose-derived mesenchymal stem cells had been a novel, minimally invasive, well-tolerated, and alternative option in the treatment of Crohn’s fistula in 2018, he said. To facilitate expanded development and application of stem cells in Crohn’s disease, we have made great progress over the past 20 years, while it is still short of reliable assessment about stem cells (SCs) in the process of fistula healing in clinic, our objective is to redefine the role of stem cells and elaborate the mechanisms after SCT in patients of Crohn’s fistula in this review.

## Material and methods

### Search strategy

We identified relevant studies by performing a comprehensive search of Pubmed and other databases (Cochrane Library and Embase) from the June 2005 to August 2020. The search was limited to clinical studies published in the English language. The search strategy was applied as below (all fields): (“mesenchymal stromal cells” OR “mesenchymal stem cells” OR “stem cells” OR “stroma cells”) AND (“fistula”) AND (“inflammatory bowel disease” OR “Crohn’s disease”), and any appropriate abbreviations.

### Study selection

All study selections were conducted by two reviewers independently without any discrepancy. Finally, they satisfied the following inclusion criteria: (1) clinical trials, (2) randomized controlled trials (RCTs) or non-randomized experimental studies (cohorts), (3) articles in English with full texts, (4) established diagnosis of CD or inflammatory bowel disease (IBD), (5) applied stem cells treatment for fistula, and (6) efficacy and/or adverse events were reported. Exclusion criteria were as follows: (1) repeated studies, (2) case reports, (3) letters or/and comments, (4) reviews or/and meta-analysis, (5) related with the pregnancy patients, and (6) non-English language articles.

### Data extraction and assessment of study quality

Eligible articles were reviewed, the data were extracted and checked, and these were performed by two reviewers in a blind manner. And any disagreement in this process was resolved by discussion. Assessment of the quality of each study included was performed using Newcastle Ottawa Quality Assessment Scale (NOS), which involves 9 items for clinical trials [40]. All answers generated the final scores for each study. A high-quality article scores 5–9 [41].

### Statistical analysis

We evaluated the degree of heterogeneity between studies using inconsistency index (*I*^2^). Generally, the values of *I*^2^ equal to 25%, 50%, and 75% were considered to indicate low, moderate, and high heterogeneity, respectively [42]. We adopted a random-effects model in our analysis. Statistical meta-analysis was performed in Review Manager 5.3 (Cochrane Collaboration, Oxford, UK) to generate forest plots of pooled risk ratio (RR), odds ratio (OR) and mean with 95% confidence intervals (CIs), and SPSS 24.0 was used to assess the efficacy (fistula healing rate) and treatment-related adverse events (TRAEs). Subgroup data was analyzed using the Chi-square test at the subgroup analysis (dose of stem cells, TRAEs), and *P* < 0.05 was considered statistically significant. Prism 6.0 was used for drawing pictures. Fistula healing, scores of Crohn disease activity index (CDAI), perianal disease activity index (PDAI), inflammatory bowel disease questionnaire (IBDQ), and level of C-reactive protein (CRP) were assessed during the follow-up.

## Results

### Search results and study characters

**Fig. 1:**
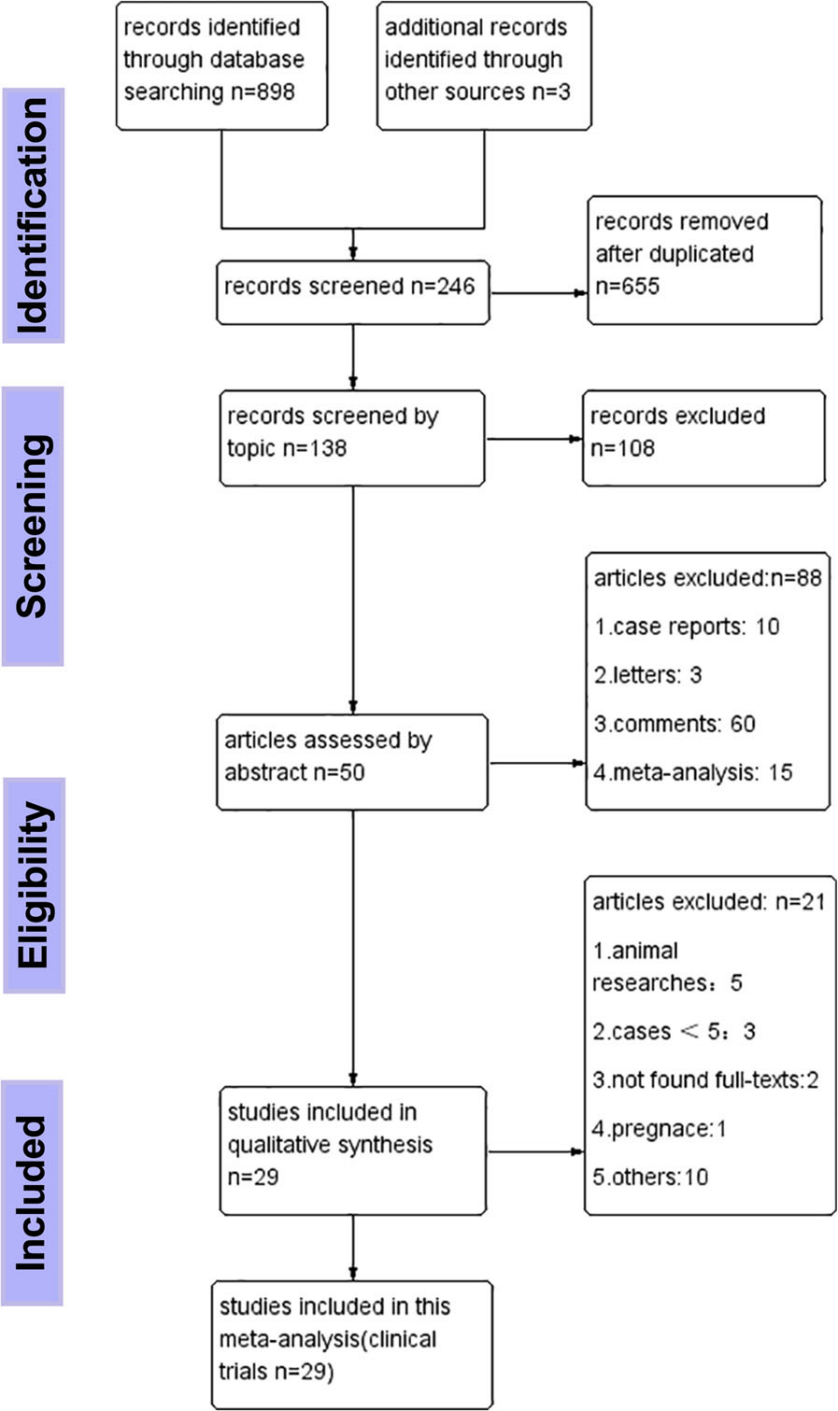
Flow chart for clinical trials of stem cells for the treatment of fistulas in this meta-analysis

A total of 901 articles were researched by our search strategy, of which 29 were included and a total of 1252 fistula patients were enrolled in our analysis by the eligibility criteria (Fig. 1), and study characteristics were shown in Table 1.There were 22 studies applied with ADSCs (four from Alofisel, Cx601, 16 from homemade cultures, one from Cx401, and one without a clear source), six with BMSCs and one with adipose tissue. The phases II–III studies accounted for approximately 44.83% (13/29) and phase I for 20.69% (6/29). Only 12 in 29 studies were RCTs: comparing applied with stem cells to placebo (e.g., Fibrin glue) [43–52], and comparing patients of Crohn’ fistula to those not with CD [43, 44, 53, 54]. The remaining received SCT with no control group. Eighteen studies used autologous stem cells [37, 44–47, 50, 55–66], seven with allogeneic stem cells [48, 49, 51, 52, 67–69], and both of them were used in other two studies [53, 54].

**Table 1:** Characteristics of clinical studies included

Author	Year of publication	Phase	Age (***M*** ± SD)	Sex (***M***:***F***)	Number of case included	Source of cell	Type of cell	Dose of cell injected	Donor	Follow-up*	Outcomes
**Both of CD and un-CD fistula**											
Garcia-Olmo et al. [43]	2009	II	43.33 ± 9.9	24:25	CD, 14 (ASC, 7; FG, 7); unCD, 35 (ASC, 17; FG, 18)	AT, Cx401	ADSC	1 × 10^7^ cells/ml	NR	7 M	FH, SF-12, SAE
Guadalajara et al. [44]	2012	II	42.6 ± 10.9	24:25	CD, 20 (ASC+FG, 17; FG, 3); unCD, 29 (ASC+FG, 7; FG, 22)	Liposuction, NR	ADSC	NR	Autologous	5 Y	FH, MRI, SAE
Garcia-Olmo et al. [53]	2015	I–II–III	49	8:2	CD, 3; un-CD, 7	AT, homemade	ADSC/SVF	1–3 × 10^7^	Autologous/allogeneic	1 Y	FH, Wexner scores, SAE
Herreros et al. [54]	2019	NR	45	24:21	CD, 18; un-CD, 24	NR, homemade	ASC/SVF	2 × 10^6^ cells/ml	Autologous/allogeneic	1 Y	FH, AE, SAE
**Only un-CD fistula**											
Herreros et al. [45]	2012	III	49.78	NR	ASC, 64; ASC+FG, 60; FG, 59	AT, homemade	ADSC	First, 2 × 10^7^ cells/ml; second, 4 × 10^7^ cells/ml	Autologous	1 Y	SF-36Q, Wexner score 22, FCS, SAE
Choi et al. [55]	2017	II	37.9	13:0	1 × 10^7^ cells/mL, 5; 2 × 10^7^ cells/mL, 8	AT, homemade	ADSC	2 × 10^7^ or 4 × 10^7^ cells/ml	Autologous	6 M	FH, Wexner scale, SAEs
Dozois et al. [56]	2019	I	39.8	7:8	15	AT, homemade	AD-MSC	NR	Autologous	6 M	FH, MRI, SAE
Topal et al. [57]	2019	NR	47 ± 13.1	8:2	10	AT, homemade	ADSC	NR	Autologous	9 M	PE, SAE
Garcia-Arranz et al. [46]	2020	III	50 ± 10	30:14	10 × 10^7^ ASC+FG, 23; FG, 21	NR, NR	ADSC	10 × 10^7^ cells/ml	Autologous	2 Y	PE, MRI, SF-12, Wexner incontinence score, SAE
**Only CD fistula**											
García-Olmo et al. [37]	2005	I	35.1 ± 2.4	2:3	5	AT, homemade	AD-MSC	1–3 × 10^7^ cells/ml	Autologous	30 M	FH, MRI, SAE
Ciccocioppo et al. [58]	2011	NR	32	8:4	12	BM, homemade	BM-MSC	NR	Autologous	1 Y	CDAI; PDAI; MRI; endoscopy; FoxP3; T cell; IL-2, 5, 10, and 12; IFN; TNF-a; SAE
Cho et al. [59]	2013	II	26. ± 6.0	4:6	1 × 10^7^ cells/ml, 3; 2 × 10^7^ cells/ml, 4; 4 × 10^7^ cells/ml, 3	AT, homemade	ADSC	1/2/4 × 10^7^ cells/ml	Autologous	8 M	FH, CD4/CD8 ratio, SAE
de la Portilla et al. [60]	2013	I/IIa	36 ± 9.0	11:13	2 × 10^7^ cells/ml, 9; 6 × 10^7^ cells/ml, 15	AT, homemade	ADSC	First, 2 × 10^7^ cells/ml; second, 4 × 10^7^ cells/ml	Autologous	6 M	MRI, PDAI, CDAI, SAE
Lee et al. [61]	2013	II	26.2 ± 5.4	30:13	42	AT, homemade	ADSC	First, 15.8 × 10^7^; second, 19.1 × 10^7^	Autologous	1 Y	FH, SAE
Molendijk et al. [47]	2015	NR	37.3	12:9	1 × 10^7^ cells/ml, 5; 3 × 10^7^ cells/ml, 5; 9 × 10^7^ cells/ml, 5; placebo, 6	BM, homemade	ADSC	1/3/9 × 10^7^ cells/ml	Autologous	2 Y	FH, MRI, PDAI, CDAI, IBDQ, CDEIS, SES-CD, SF-36, CRP, IL-8, IL-1β, IL-6 and 10, TNF, IL-12p70, SAE
Ciccocioppo et al. [62]	2015	NR	39.25 ± 14.32	5:3	8	BM, homemade	BM-MSC	NR	Autologous	6 Y	MRI, CDAI, PDAI, SAE
Cho et al. [63]	2015	II	26.2 ± 5.5	28:13	41	AT, homemade	ADSC	3 × 10^7^ cells/ml	Autologous	2 Y	FH, AE, SAE
Park et al. [67]	2016	NR	32.17 ± 7.96	4:2	1 × 107 cells/ml, 3; 3 × 10^7^ cells/ml, 3	AT, homemade	ADSC	1 × 10^7^ cells/ml, 3 × 10^7^ cells/ml	Allogeneic	6 M	FH, MRI, CD4/CD8 ratio, SAE
Panés et al. [48]	2016	III	38	116:96	ASC, 107; placebo, 105	AT, alofisel	AD-MSC	12 × 10^7^ cells	Allogeneic	6 M	MRI, PDAI, CDAI, IBDQ, van Assche score, IgG AE, SAE
García-Arranz et al. [68]	2016	I–IIa	35	NR	10	AT, homemade	ADSC	First, 2 × 10^7^ cells; second, 4 × 10^7^ cells	Allogeneic	4.5 M	QoL, SF-36, fecal incontinence, severity index
Dietz et al. [64]	2017	I	35 ± 14.21	6:6	12	BM, homemade	MSC	2 × 10^7^ cells/ml	Autologous	6 M	MRI, Van Assche score, SAE
Scott [69]	2018	III	38	NR	24	AT, alofisel	ADSC	12 × 10^7^ cells	Allogeneic	13 M	IL-6, 12, and 10; TNF-α; TGF-β; PDAI; CDAI; SAE
Panés et al. [49]	2018	III	38.3	114:98	12 × 10^7^ cells, 107; placebo, 105	AT, alofisel	ADSC	12 × 10^7^ cells	Allogeneic	13 M	IBDQ, PDAI, CDAI, TNF, MRI, TEAE
Wainstein et al. [70]	2018	NR	36	2:7	9	AT, homemade	ADSC	NR	Autologous	37 M	IBDQ, PDAI, SAE
Avivar-Valderas et al. [52]	2019	NR	NR	NR	ASC 58; placebo 42	AT, Alofisel	AD-MSC	NR	Allogeneic	13 M	DSA; HLA-I; CRPs; CD55, 46, and 59; AE; SAE
Dige et al. [66]	2019	NR	NR	6:15	21	AT, homemade	AT	NR	Autologous	6 M	FH, complications
Zhou et al. [50]	2020	NR	28.86 ± 10.13	21:1	ADSC, 11; placebo, 11	AT, homemade	ADSC	5 × 10^6^ cells/ml	NR	1 Y	FH, MRI, ultrasonography, CDAI, PDAI, IBDQ, VAS, Wexner score, CRP, ESR, FC, SAE
Barnhoorn et al. [51]	2020	NR	42	8:6	1 × 10^7^ cells/ml, 5; 3 × 10^7^ cells/ml, 5; 9 × 10^7^ cells/ml, 5; placebo, 6	BM homemade	BM-MSC	1/3/9 × 10^7^ cells/ml	Allogeneic	4 Y	FH, HLA, MRI, rectoscopy, PDAI, CDAI, Vaizey, QoL, SF36, IBDQ, SAE
Lightner et al. [65]	2020	NR	49	0:5	5	BM, homemade	MSC	NR	Autologous	6 M	FH, MRI, AE, SAE

*Y = years and M = months

Abbreviations: *NR* not report, *FG* fibrin glue, *AT* adipose tissue, *BM* bone marrow, *ADSC* adipose-derived stem cells, *SVF* stromal vascular fraction, *MSC* mesenchymal stem cell, *BM*-*MSC* bone marrow-derived mesenchymal stromal cells, *FH* fistula healing, *PE* physical examination, *QoF* quality of life, *MRI* magnetic resonance imaging, *FCS* fistula complexity score, *CDAI* Crohn’s disease activity index, *PDAI* perianal disease activity index, *CDEIS* Crohn’s disease endoscopic index of severity, *SES*-*CD* simplified endoscopic activity score, *CRP* C-reactive protein, *TGF*-*β* transforming growth factor-β, *DSA* donor-specific antibodies, *VSA* pain scores with visual analog score, *ESR* erythrocyte sedimentation rate, *FC* fecal calprotectin, *GF* growth factor, *BLI* bioluminescence, *TEAEs* treatment-emergent adverse events

### Quality assessment

The qualities of included studies were assessed by using the NOS, and we analyzed 29 clinical studies enrolled and finally had a total score of 175 with a mean of 6 and a range of 3 to 9 for each study (Supplement Table 1). In total, the quality of clinical trials reached the “high-quality study” level.

### Primary outcomes

#### Efficacy of stem cells therapy for Crohn’ fistulas

**Fig. 2:**
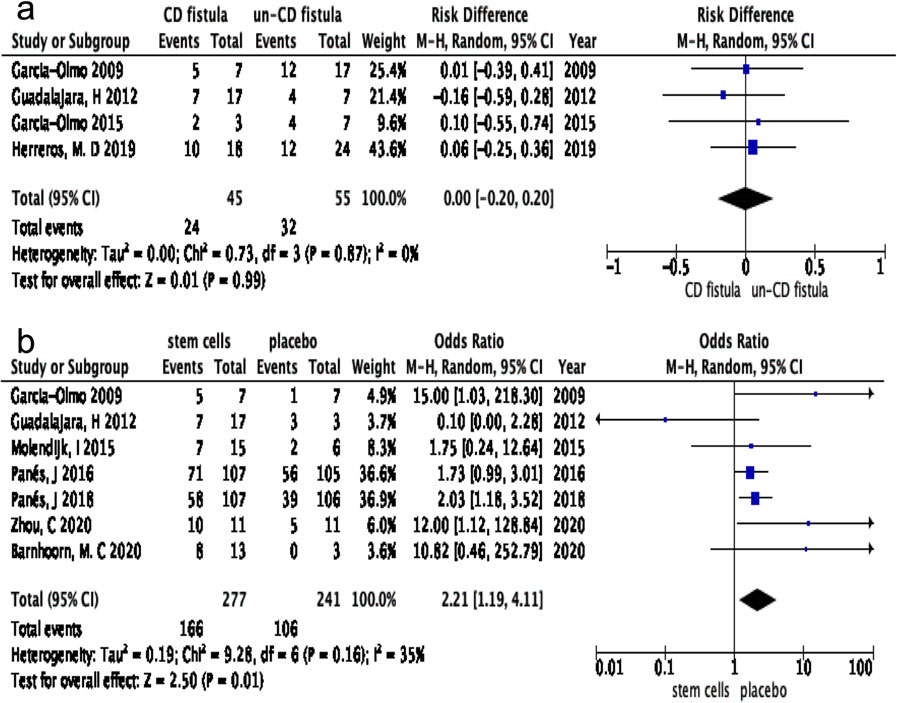
**a** CD fistulas versus un-CD fistulas for fistula healing administrated by stem cells. **b** Stem cells versus placebo administrated for fistula healing in the treatment of Crohn’s fistula

Four of 12 RCTs reported fistula healing in Crohn’s fistula and un-CD fistula [43, 44, 53, 54]. Seven reported fistula healing administrated with stem cells and placebo (such as fibrin glue) [43, 44, 47–51]. The random-effects model was used to assess the differences in healing rate (HR) between the experiment group (Crohn’s fistulas, stem cell group) and control group (un-CD fistulas, placebo group). Regardless of the dose of stem cells injected and route of administration, the HRs of fistula in patients with CD and without CD were 58.71% and 58.72%, respectively (*I*^2^ = 0%, RR 0.00, 95% CI − 0.20 to 0.20, *P>0*.*05*) (Fig. 2a). Moreover, patients with Crohn’s fistula in the stem cell group had a higher HR of 61.75% than in the placebo group (40.46%). There was a significant statistical difference (*I*^2^ 35%, RR 2.21, 95% CI 1.19 to 4.11, *P* = *0*.*01*) (Fig. 2b). As for the source of stem cells, HR of Cx601 administration in patients of Crohn’s fistula was about 61.02%, which was higher than of ASCs from homemade cultures (51.43%, *P < 0*.*05*).

#### The doses of stem cells injected

**Fig. 3:**
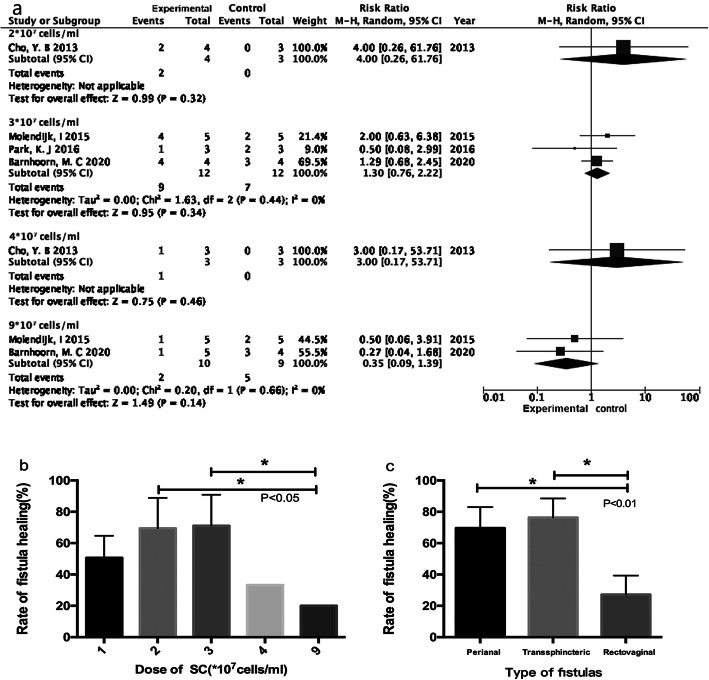
**a** Forest plot of doses of stem cells implanted for Crohn’ fistula. **b** Fistula healing rate of dose of stem cells implanted for Crohn’s fistula. **c** Fistula healing rate of different fistula type administrated by stem cells

There were four clinical trials involving the different doses of SCT [47, 51, 59, 67], and a total of 31 patients of Crohn’s fistula received the different doses of stem cells. They were divided into five subgroups: 1 × 10^7^ cells/mL, 2 × 10^7^ cells/mL, 3 × 10^7^ cells/mL, 4 × 10^7^ cells/mL, and 9 × 10^7^ cells/mL groups, respectively. The patients injected with the dose of 1 × 10^7^ cells/mL group were defined as the control group, and others were experimental groups. As Fig. 3a showed, only one study was treated with stem cells of doses of 2 × 10^7^ cells/mL (*n* = 7) and 4 × 10^7^ cells/mL (*n* = 6), which was lack of completely valid data [59], and three studies with stem cells of dose of 3 × 10^7^ cells/mL (*n* = 24, [47, 51, 67]), two with 9 × 10^7^ cells/mL (*n* = 19, [47, 51]). The dose of 3 × 10^7^ cells/mL group had a stronger advantage in Crohn’ fistula healing compared to 1 × 10^7^ cells/mL group (*I*^2^ 0.00%, OR 1.30, 95% CI 0.76 to 2.22), but the dose of 9 × 10^7^ cells/mL group was defeated by the 1 × 10^7^ cells/mL group in terms of fistula healing (*I*^2^ 0.00%, OR 0.35, 95% CI 0.09 to 1.39). The HRs of patients with Crohn’s fistula in 1 × 10^7^ cells/mL, 2 × 10^7^ cells/mL, 3 × 10^7^ cells/mL, 4 × 10^7^ cells/mL, and 9 × 10^7^ cells/mL group were 50.60%, 69.50%, 71.00%, 33.00%, and 20.00%, respectively (showed in Fig. 3b). Compared to 4 × 10^7^ cells/mL and 9 × 10^7^ cells/mL groups, there were statistically significant differences in 2 × 10^7^ cells/mL and 3 × 10^7^ cells/mL groups, respectively (both of *P < 0*.*05*). Overall, the dose of 3 × 10^7^ cells/mL stem cells had a higher superiority in patients of Crohn’s fistula.

#### The types of Crohn’s fistula

Nine of 29 studies reported HRs based on fistula types with a total of 99 fistulas [37, 43, 44, 54, 64, 66–68, 70]. They mainly comprised perianal, transsphincteric, and rectovaginal fistulas. Our analysis involved in perianal fistula with seven studies, transsphincteric fistula with three studies, and rectovaginal fistula with seven. As Fig. 3c showed, the patients with perianal and transsphincteric fistulas had more apparent HRs compared to those with rectovaginal fistulas (77.95%, 76.41% vs. 27.18%, *P < 0*.*01*).

### Secondary outcomes

#### Related scores and indicator assessment

**Fig. 4:**
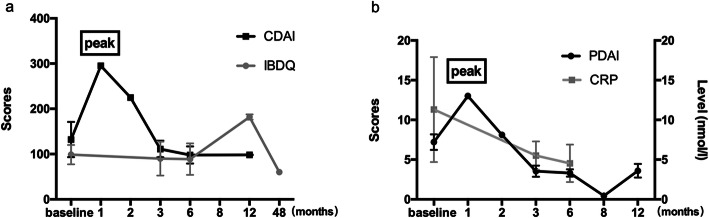
Different assessment index at follow-up. **a** CDAI and IBDQ. **b** PDAI and CRP

Most of clinical studies reported the detailed changes of scores and lab indicators, such as CDAI, PDAI, IBDQ, and CRP. Of them, four studies reported variation of CDAI after administrating stem cells [47, 49, 58, 60], seven articles were referred to changes of PDAI [47–50, 58, 60, 70], five to scores of IBDQ [47, 49–51, 70], and two to the level of CRP [47, 50]. For scores of CDAI, 1 month later, it occurred a transient rise among patients with the treatment of stem cell (295.00 vs. 132.10, *P < 0*.*05*), while 3 months later, it returned to the baseline score and last to endpoint (295.00 vs. 111.14, *P < 0*.*05*) (Fig. 4a). It had a mild difference in the variation of PDAI score—there were two rising tendency after 1 month and 12 months by SCT, respectively, and it downed to 3.54 and 3.31 until 3 and 6 months after giving SCs. There was a statistical significant difference between pre-stem cells and post-stem cells (*P < 0*.*05*) (Fig. 4b). Besides that, scores of IBDQ did not increase until 6 months later by SCT and reached a peak at 12 months with no significance difference (88.85 ± 69.96 vs. 181.95 ± 8.27, *P > 0*.*05*). Moreover, after stem cells therapy, the level of CRP (nmol/l) showed continuous drops (baseline vs. 6 months, 11.3 ± 13.23 vs. 4.5 ± 4.7, *P < 0*.*05*). These details could be found in Figs. 4a and b.

#### TRAEs analysis

**Fig. 5:**
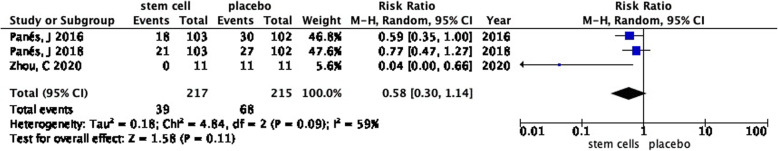
Forest plot of TRAEs between stem cells and placebo in patients with Crohn’s fistula

All of studies showed the adverse events (AEs) and severe adverse events (SAEs). However, in terms of TRAEs, the details and accurate data are only reported in three RCTs [48–50] and eight cohort studies. Figure 5 showed the difference of the incidence rate of TRAEs between the stem cell group and placebo group, patients in the stem cell group had an advantage over in placebo group (*I*^2^ 59%, RR 0.58, 95% CI 0.30 to 1.14). In cohort studies, only studies by de la Portilla et al. and Scott et al. [60, 69] reported five TRAEs.

## Discussion

To our knowledge, this review summarized that SCT had a higher efficacy and lower incidence of adverse events in patients with Crohn’s fistula compared with conventional therapies. We revealed that the rate of fistula healing ranged from 58 to 62% after administrating SCs in patients of Crohn’s fistula, which was similar to our previous results with a clinical remission of 62.52% [39]. What is more, Cx601 (darvadstrocel) administration in patients of Crohn’s fistula had a higher HR of 61.02% compared to homemade cultures, it had strongly evidenced that it was necessary and important for a systematic, maturing and professional protocol in clinical work. Our analysis also found, whether patients suffered Crohn’s fistula or not (un-CD fistula), the HR had no statistically significant difference (58.71% vs. 58.72%). Considering the properties of immunoregulatory, anti-inflammatory, and multipotential differentiation of stem cells [33, 34, 71–73], which one dominated the important position and what the potential mechanism was? The possible explanations were presented as follows: primarily, SCs could alter immune reconstitution by stimulating de novo generation of an altered T cell repertoire and eliminating aberrant clones, it then would replace fistulas [74]. Secondly, SCs could release various mediators and multiple metabolites, such as growth factors, exosomes, and chemokines, to repair injured tissue and promote its regeneration by strengthening differentiation [75]. Plus, the study by Clevers et al. also had referred to “niche” of stem cells, and it was able to promote self-organization of multiple mammalian tissues by the self-renewal factors of Wnt signaling [76]. Conversely, stem cells’ fate was influenced by various growth factors to differentiate into normal tissues [77]. Obviously, the latter match more with roles of stem cells in this process among patients of Crohn’s fistula and un-CD fistula. It was, therefore, worth supposing that stem cells might work in patients of Crohn’s fistula and un-CD fistula by means of multi-directional differentiation to achieve tissue repair, rather than of immune reconstitution only.

With respect to the doses of SCT in patients of Crohn’s fistula in our review, data on the use of stem cells at different doses was limited. And a variety of articles consistently demonstrated that an optimal dose was the key point in the protocol of stem cells for Crohn’s fistula and was a focused problem for a long time in clinical and pre-clinical studies. In this study, only two clinical studies by Molendijk et al. and Barnhoorn et al. [47, 51] randomly assigned CD patients to three groups based on doses of stem cells: 1 × 10^7^ cells/mL, 3 × 10^7^ cells/mL, and 9 × 10^7^ cells/mL, and they demonstrated that 3 × 10^7^ cells/mL group had the highest HR. Meanwhile, the study of Scott reported that a single dose of 120 million stem cells (Darvadstrocel) administered into the perianal fistulas tissue was significantly more effective than placebo (saline) with a clinical remission of more than 50% [69]. Additionally, our published articles had put forward that the best range of dose of stem cells in Crohn’s fistula was 2–4 × 10^7^ cells/mL with a HR of up to 80.07% [39]. Surprisingly, we found that stem cells at the dose of 3 × 10^7^ cells/mL had the highest HR of 71.00% compared to other doses of cells in this meta-analysis, which was beneficial for improving the efficacy in Crohn’s fistula at a large degree and narrowed down the optimal dose of SCs further.

Given the classification of fistulas, they could be divided into simple and complex fistulas based on American Gastroenterological Association classification system [78, 79], as well as into intersphincteric, transsphincteric, extrasphincteric, or suprasphincteric fistulas by anatomy [80]. In our study, it referred mostly to perianal, rectovaginal, intersphincteric, and transsphincteric fistulas. Only nine of them reported the fistula healing rate based on the fistula types. Surprisingly, we noticed that patients of perianal and transsphincteric fistulas had a better reaction to SC implantation than of rectovaginal fistula with the efficacy of 77.95% and 76.41%, respectively. But the reason was not lucid. Among patients of perianal fistulas, the inflammation was extremely severe due to roles of intestinal bacterial and communication of excrement and perianal skin surface to trigger an immune response and cytokine production [43, 81]. As the study by Cellerix et al. supported, SCs could be stimulated by relatively high concentrations of pro-inflammatory cytokines (IFN-γ) produced by fistula lesions, and expressed indoleamine 2, 3-dioxygenase (IDO) of metabolizing tryptophan to kynurenine, which had an anti-inflammatory effect. So, let us make a hypothesis that in the relatively severe inflammatory microenvironment of perianal area, SCs play a remarkable role of anti-inflammatory, which was consistent with what we discussed previously (the “Discussion” section, paragraph 1, [82, 83]). Meanwhile, the second reason, perianal and transsphincteric fistulas themselves were also believed to own a relatively dry and comfortable local anatomical circumstance, which contribute to renovate impaired tissue being different from rectovaginal fistulas [54].

CDAI was defined to evaluate the efficacy and severity of Crohn’s disease, as well as PDAI [84]. In 2017, our study already showed that there was a different clinical remission between patients of CDAI baseline > 150 group and < 150 group by contrasting the change of level of CDAI after SCT [39]. While it did not refer to the change of CDAI at each time point—we solved it in this review. Coincidentally, we found a transient rise at 1 month extensively and reduced to a level below the baseline at 3 months after injecting SCs. Which of the mechanisms were playing significant roles in it? An animal experiment might have mirrored our results: once stem cells were injected into fistula walls among rats of Crohn’s fistula, effective stem cell mass was reduced from inception, and the detectable cells represented only 8.87% of their initial amount to take action at the first 30 days via monitoring the dynamics of bioluminescence (BLI) [85]. Moreover, the study of TiGenix et al. [86] had confirmed that ADSCs were present in the rectum and jejunum for ≥ 14 days and undetectable after 3 or 6 months in any of the tissues. Taken together, we can hypothesize that at the period of 1 month after SCT, up to 90% of stem cells vanished and destroyed by peoples’ immune clearance themselves; after 3 months, the remaining SCs (about 8%) were differentiated into functional epithelial cells or stromal cells; meanwhile, the phases of immune tolerance and reconstruction started up [74]. The defined and powerful mechanisms need further pre-clinical and clinical studies.

### Limitations

Several limitations of our meta-analysis should be acknowledged: (1) In terms of animal researches, they were small-sized and lacked detailed data about mucous healing; assessment index, such as IL-2, IL-6, IL-8, and IL-10; and the adverse events, which failed to analyze systematically. (2) In clinical trials, they did not report the results about immunohistochemistry and endoscopy in quantification. (3) Subgroup analysis was inadequate owing to the majority of studies included about cohort studies. (4) Given the limited follow-up among included studies, we failed to elaborate the recurrent after administrating stem cells among CD patients. (5) We used random-effects model to account for the statistical heterogeneity conservatively in the pooled studies.

## Conclusions

The utility of stem cells in patients of Crohn’s disease is a potential way, but still in the very early stages, particularly in Crohn’s fistula; the achievements are encouraging now but not comprehensive and systematic. Based on our review, SC treatment in treatment of Crohn’s fistula has a higher efficacy (fistula healing rate), as well as a lower TRAEs compared to other options. And the optimal dose of 3 × 10^7^ cells/mL SCs injected has been determined. While a gold standard is still not be identified, we are eager to do more basic and clinical studies performed to further ascertain protocol and break current dilemmas.

## Supplementary Information

**Additional file 1.**

## Data Availability

The data supporting the conclusions of this article are all online.

## Abbreviations

SCs: Stem cells
SCT: Stem cell therapy
RR: Risk ratios
OR: Odds ratio
CIs: Confidence intervals
ADSCs: Adipose-derived stem cells
RCTs: Random control trials
HR: Healing rate
CD: Crohn disease
IBD: Inflammatory bowel disease
5-ASA: 5-Aminosalicylic acids
6-MP: 6-Mercaptopurine
TNFs: Anti-tumor necrosis factors
SF-36: MOS item short from health survey score
BMSCs: Bone marrow-derived stem cells
NOS: Newcastle Ottawa Quality Assessment Scale
TRAEs: Treatment-related adverse events
CDAI: Crohn disease activity index
PDAI: Perianal disease activity index
IBDQ: Inflammatory bowel disease questionnaire
CRP: C-reactive protein
AEs: Adverse events
SAEs: Severe adverse events
