# Supplementary material for: Efficacy of stem cells therapy for Crohn’s fistula: a meta-analysis and systematic review
Source: Stem Cell Res Ther. 2021 Jan 7;12:32. doi: 10.1186/s13287-020-02095-7 (PMC7792029; doi:10.1186/s13287-020-02095-7)
Supplement: Supplementary file 1 — Additional file 1. [file 13287_2020_2095_MOESM1_ESM.docx]

**Supplement table 1 Quality assessment based on the Newcastle-Ottawa Scale of studies enrolled**

| **Author** | **Year** | **Representativeness of the exposed cohort** | **Selection of the non-exposed cohort** | **Ascertainment of exposure** | **No-demonstration of interesting outcome at start of study** | **Control for important factor or additional factor** | **Assessment of outcome** | **Enough follow-up of outcome** | **Adequacy of follow up of cohorts** | **Total quality scores** |
| --- | --- | --- | --- | --- | --- | --- | --- | --- | --- | --- |
| Zhou, C. | 2020 | 1 | 1 | 1 | 1 | 1 | 1 | 1 | 0 | 7 |
| Barnhoorn,M. C | 2020 | 1 | 1 | 1 | 1 | 0 | 1 | 0 | 0 | 5 |
| Lightner, A. L | 2020 | 1 | 0 | 1 | 0 | 0 | 1 | 0 | 0 | 3 |
| Garcia-Arranz,M | 2020 | 1 | 1 | 1 | 0 | 1 | 1 | 0 | 1 | 6 |
| Avivar-Valderas,A | 2019 | 1 | 0 | 1 | 1 | 0 | 1 | 0 | 0 | 4 |
| Dige, A | 2019 | 1 | 0 | 1 | 1 | 0 | 1 | 1 | 0 | 5 |
| Dozois, E. J | 2019 | 1 | 0 | 1 | 0 | 0 | 1 | 1 | 0 | 4 |
| Topal, U. | 2019 | 1 | 0 | 1 | 1 | 0 | 1 | 1 | 1 | 6 |
| Herreros, M. D | 2019 | 1 | 1 | 1 | 1 | 2 | 1 | 1 | 1 | 9 |
| Scott, L. J | 2018 | 1 | 0 | 1 | 1 | 0 | 1 | 1 | 1 | 6 |
| Panés, J | 2018 | 1 | 1 | 1 | 1 | 1 | 1 | 1 | 1 | 8 |
| Wainstein, C | 2018 | 1 | 0 | 1 | 0 | 0 | 1 | 0 | 0 | 3 |
| [Dietz, A. B](http://www.ncbi.nlm.nih.gov/pubmed/?term=Garcia-Olmo%2520D%255bAuthor%255d&cauthor=true&cauthor_uid=19273960) | 2017 | 1 | 0 | 1 | 1 | 0 | 1 | 1 | 1 | 6 |
| Choi, S | 2017 | 1 | 1 | 1 | 1 | 2 | 1 | 1 | 1 | 9 |
| Park, K. J | 2016 | 1 | 1 | 1 | 1 | 1 | 1 | 1 | 1 | 8 |
| Panés, J | 2016 | 1 | 1 | 1 | 1 | 1 | 1 | 1 | 1 | 8 |
| García-Arranz, M | 2016 | 1 | 0 | 1 | 0 | 1 | 1 | 1 | 1 | 6 |
| García-Arranz, M | 2015 | 1 | 0 | 1 | 1 | 1 | 0 | 1 | 1 | 6 |
| Ciccocioppo, R. | 2015 | 1 | 0 | 1 | 0 | 1 | 1 | 1 | 1 | 6 |
| Cho, Y. B. | 2015 | 1 | 0 | 0 | 0 | 1 | 1 | 1 | 1 | 5 |
| Molendijk, I | 2015 | 1 | 1 | 1 | 1 | 1 | 1 | 1 | 1 | 8 |
| Cho, Y. B | 2013 | 1 | 1 | 1 | 1 | 1 | 1 | 1 | 0 | 7 |
| Lee, W. Y | 2013 | 1 | 0 | 1 | 0 | 1 | 1 | 0 | 1 | 5 |
| de la Portilla, F | 2013 | 1 | 1 | 1 | 1 | 1 | 1 | 0 | 1 | 7 |
| Guadalajara, H | 2012 | 1 | 1 | 1 | 1 | 1 | 1 | 1 | 1 | 8 |
| Herreros, M. D | 2012 | 1 | 1 | 1 | 0 | 1 | 1 | 0 | 1 | 6 |
| Ciccocioppo R | 2011 | 1 | 0 | 0 | 1 | 0 | 0 | 1 | 1 | 4 |
| Garcia-Olmo | 2009 | 1 | 1 | 1 | 0 | 1 | 1 | 1 | 1 | 7 |
| García-Olmo | 2005 | 1 | 0 | 0 | 1 | 0 | 1 | 0 | 0 | 3 |

Total score, 9; <=4, poor quality；> 4, good quality.
